# Cultivation and Uses of *Moringa oleifera* as Non-Conventional Feed Stuff in Livestock Production: A Review

**DOI:** 10.3390/life13010063

**Published:** 2022-12-25

**Authors:** Khalid Abdoun, Ahmed Alsagan, Osman Altahir, Gamaleldin Suliman, Ahmed Al-Haidary, Mohammed Alsaiady

**Affiliations:** 1 Department of Animal Production, College of Food and Agriculture Sciences, King Saud University, P.O. Box 2460, Riyadh 11451, Saudi Arabia; 2 King Abdulaziz City for Science and Technology, Riyadh 12354, Saudi Arabia; 3 Biostatistics Department, Naif Arab University for Security Sciences, Riyadh 14812, Saudi Arabia; 4 Development and Research Unit, Arabian Agricultural Services Company (ARASCO), Riyadh 12311, Saudi Arabia

**Keywords:** *Moringa oleifera*, botanical practices, nutritional potential, livestock nutrition

## Abstract

*M. oleifera* is the best known and the most utilized of the 14 known species of the genus Moringa. Moringa is used as animal fodder and a medicinal plant as well as in the purification of water. Studies have shown that the day/night temperature of 30/20 °C is the most favorable for *M. oleifera* germination, plant growth and development. *M. oleifera* plants prefer sandy, well-drained loam soils due to their susceptibility to waterlogged soil conditions. It is recommended to use fertilizers to improve plant growth and the amount of forage production in areas with low rainfall and extreme temperatures. For forage production, an area of 20 × 20 cm is adapted to 16,000 plants per hectare. Chemical analyses confirmed the presence of different groups of pharmacologically active chemical compounds, as well as functional compounds of nutritional value such as carbohydrates, proteins, fats and vitamins, in *M. oleifera*. The reviewed literature particularly encourages the use of *M. oleifera* whole plants as nonconventional forage in ruminants’ nutrition, as well as using *M. oleifera* leaves or leaves extract as a protein source for broilers and laying hens. *M. oleifera* in livestock feed with the ultimate goal of producing functional food (meat, eggs and milk) with appropriate contents of human health-promoting substances such as omega-3 and organic selenium remains to be elucidated. Furthermore, *M. oleifera* inclusion in livestock feed has the potential to increase the shelf-life of animal products during storage and processing. Further research is needed to determine the appropriate supplementation level of different plant parts or their extracts, as well as the appropriate processing methods or treatments of *M. oleifera,* in order to improve its palatability and consequently enhance the production performance of livestock without compromising animal health.

## 1. Introduction

Moringa belongs to the family Moringaceae which grows mainly in tropical countries and is a drought-tolerant species. There are 14 known species of the genus Moringa, of which *M. oleifera* is the most widely distributed, the best known and the most utilized. The species range from small herbaceous plants to big trees. Moringa species tolerate a large number of environmental stresses; it resists up to 48 °C and the tree can grow well in regions that receive 250–1500 mm annual rain fall [1]. Large numbers of studies on the Moringa plant’s nutritional qualities exist in both scientific and popular literature. There are many known uses of this tree; it can be used as a source of food, fodder, medicine, plant growth stimulants, cosmetics, water purification and biofuel production [2,3,4,5,6].

It is a tree with noticeable environmental and economic importance. It is a plant that does not need much care and is rich in bioactive compounds. This species has also been shown to present some tolerance to water deficit and environmental stresses. Almost all parts of the *M. oleifera* tree contain essential nutrients such as proteins, carbohydrates, vitamins (A, B1, B2, B3, C and E) as well as minerals [7,8,9]. The leaves and roots have a high content of total phenols, tannins and flavanols [3,10]. Marcu [11] claimed that there are no negative effects from the daily consumption of *M. oleifera* leaves. The seeds and leaves of *M. oleifera* are usually eaten fresh, cooked or added to other types of foods in a powdered form for nutritional purposes [2,12]. Therefore, *M. oleifera* is a good tree that can be used globally to combat nutritional deficiencies and poverty [5,13].

Thus, Moringa is a promising biotechnological tree; it has a high number of uses and it is a source of a number of vital biological compounds. Recent studies show that *Moringa oleifera* dried leaves are characterized by higher crude protein, vitamin C, β-Carotene, Se, total phenol and antioxidant activity and lower fiber contents compared to alfalfa hay [14,15,16].

## 2. Botanical and Cultural Pract Ices of *Moringa oleifera*

This subsection will overview the botanical and cultural practices concerning *M. oleifera* including the suitable environmental conditions for its cultivation as well as its soil, fertilizer and water requirements. In addition, cultivation density, growth rate, seeds storage conditions and the chemical composition of *M. oleifera* will be reviewed.

### 2.1. Cultivation Condition and Requirements

Moringa is a small, slender, fast-growing, deciduous shrub or tree 9 to 15 m in height, with an open canopy-shaped crown. Moringa is of exceptional nutritional value with a variety of medicinal properties and grows well in a wide range of soils but deep sandy loam soil with a pH of 6.5–8.0 is the best [2,17]. Usually, *M. oleifera* seeds are sown directly in the field or by seedlings that are raised in nurseries [18]. Among the environmental factors that affect plant growth, temperature is the most important factor that controls the natural geographic distribution, trees and shrubs performance, physiology and productivity of *M. oleifera* [2]. Studies have also shown that the day/night temperature of 30/20 °C is the most favorable for *M. oleifera* germination, plant growth and development, and it has also been emphasized that tropical and subtropical environmental conditions with hot summers and mild winters are favorable for cultivation of *M. oleifera* [3,19]. According to Manduwa et al. [20], the performance of the flowering stage of *M. oleifera* depends on the temperature. Temperatures between 30 and 35 °C promote fruit formation while lower temperatures (below 15 °C) result in a reduced rate of fruit growth in the flowering period. Although high temperatures are very suitable for *M. oleifera* growth, low but satisfactory growth and reasonable yield can still be achieved in sub-optimal environments [21]. Successful cultivation of *M. oleifera* in cold environments will greatly increase its yield; however, the impact of cultivation under cold environments should be well understood before planting.

*M. oleifera*, which is a high temperature-suited plant species, grows best with annual precipitation between 250 mm and 1500 mm [2]. Physiological processes such as photosynthesis, growth and respiration are controlled by metabolic pathways that are influenced by temperature and its seasonal changes [22]. Moreover, gas exchange is affected by seasonal changes [23]. In summer, an increase in the level of CO_2_ uptake across the stomata was observed, while transpiration, stomata conduction and the rate of photosynthesis decreased. *M. oleifera* plants reduce these traits as an adaptive mechanism and increase water use efficiency under lower rainfall and higher temperatures. This is due to the plant’s ability to store carbon in its succulent parts during growth periods [23]. *M. oleifera*’s CO_2_ absorption rates are 20 times higher than those of other plants. Moreover, *M. oleifera* trees are highly susceptible to frost and cold environmental conditions [18].

**Figure 1:**
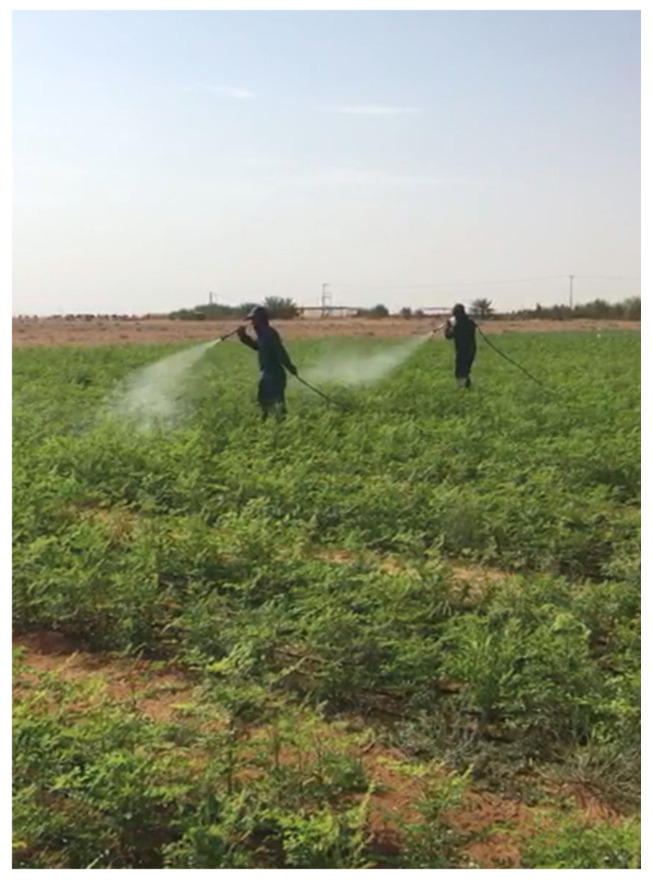
Foliar spray of *M. oleifera* by Amino Se fertilizer.

Fertilization of *M. oleifera* grown in sandy soil with NPK resulted in maximum growth and high leaf yield with good nutrient and biochemical attributes [24,25]. *M. oleifera* annual yield can reach up to 27 tons of dry matter per hectare at a planting density of 167,000 plant/ha, when fertilized by N at a level of 521 kg/ha/year [26]. Furthermore, nitrogen fertilization seems to affect the chemical composition of *M. oleifera* by increasing its protein content [27]. Recently, foliar spray of *M. oleifera* by selenium fertilizer has been practiced to fortify its selenium content [28] (Figure 1).

In arid and semi-arid regions of the tropics, *M. oleifera* leaves are shed during the dry season and resume leaf regrowth during the rainy season [1]. There is a prevailing belief that pruning enhances the growth of the vegetative parts, including leaves, at high rates, which leads to a dense growth of additional leaves and lateral buds. Without pruning, the tree may produce an upright, fast-growing shoot with only limited leaves and fruits on the main stem [1]. Pruning of *M. oleifera* was recommended to enhance branching, increase yield and facilitate harvesting [29]. Vijayakumar [30] claimed that early pruning of Moringa plants, which was conducted after 60 days of sowing, is better than pruning after 90 days of sowing as it increased the growth rate and had higher forage yield.

Light and medium pruning helps increase canopy size and leaf growth compared to heavy pruning. The potential low carbohydrate supplies due to the low leaf surface of heavily pruned trees did not provide enough photosynthesis to allow significant increases in the vegetative growth. A physiological stimulating effect of moderate pruning on tree growth rates was observed [31].

### 2.2. Plant Density, Production and Storage Conditions

**Figure 2:**
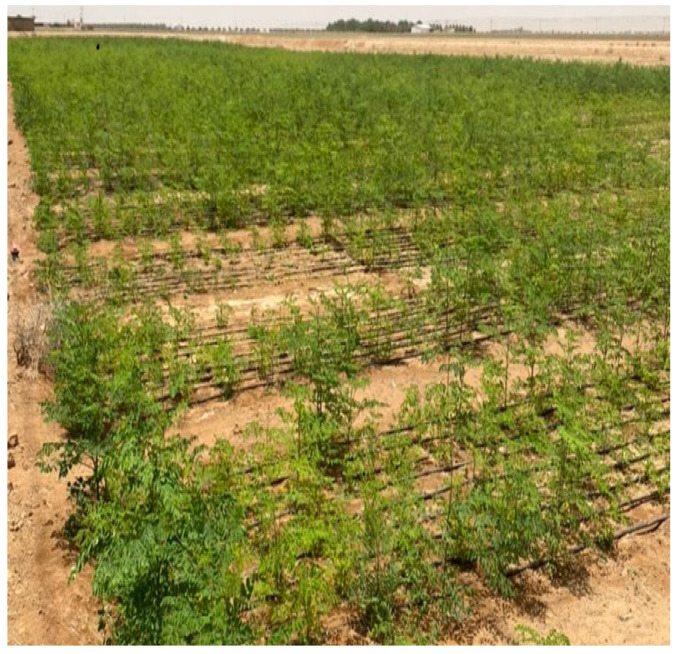
Mass production of *M. oleifera* as a forage.

Studies have indicated the use of 5000 plants/hectare for planting *M. oleifera* under favorable conditions for the stages of flower growth and *M. oleifera* fruiting. The sowing date should be strictly adhered to as the flowering stage should not intersect with the monsoon season which may lead to heavy flower shedding. For forage production, an area of 20 × 20 cm is adapted to 16,000 plants per hectare, pits of 10 × 10 × 10 cm are dug and then the seeds are sown in the center of the pit (Figure 2).

**Figure 3:**
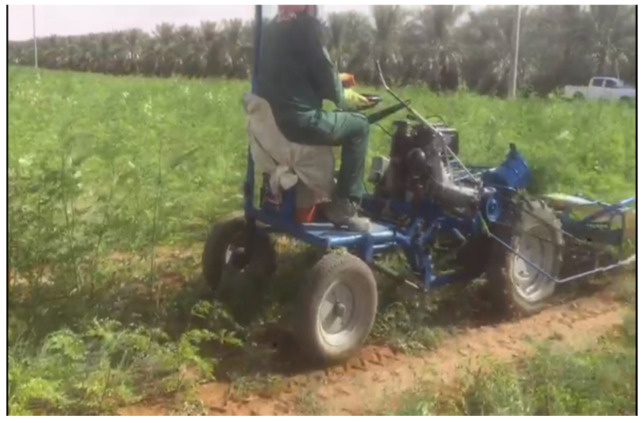
Mechanized harvesting of *M. oleifera*.

The seed germinates 10 to 12 days after sowing. The seed requirement per hectare is 6 kg/ha. Pre-treatment of Moringa seeds with Azospirillum inoculum at a rate of 100 gm per 6 kg of seeds increased the germination rate and increased growth rates and productivity. However, it was found that the tree is sensitive to the intensity of pruning or its absence [32]. For mechanized harvesting (Figure 3), *M. oleifera* can be harvested at a height of 0.5 m above the soil surface when the stem is relatively soft [33]. Storage, temperature, moisture content and seed age are key factors that affect the viability and quality of Moringa seeds [33]. Moringa seeds can be stored inside paper or aluminum bags for up to 6 months, after which time they should be stored in closed containers at temperatures between 4 and 19 °C [34]. Farmers are advised to store their seeds at temperatures below 20 °C in paper bags for up to 12 months, and the moisture content of the seeds should be less than 8% [35]. Another way to store seeds is to store them in their fruits in dry, cool conditions for up to 12 months. Seeds can also be dried for 30 days before being stored in airtight containers to ensure high quality seeds [36]. Moringa leaves can be transported and consumed throughout the year without a decrease in the quality of nutrients [37].

### 2.3. Chemical Composition of Moringa oleifera

The nutritional content of *Moringa oleifera* showed significant differences which were attributed to genetic factors, environmental factors and cultural practices [38]. *M. oleifera* seeds are the best natural anticoagulant that have antimicrobial and antioxidant properties and are efficiently used to treat and purify highly turbid water [2,39]. The seeds also contain oil that is of high nutritional quality and can be used for cooking [40,41]. *M. oleifera* leaves can be used as food which is available all year round and is of high quality. The young leaves are edible and are usually consumed after cooking or prepared as soup or salad [42].

The chemical analyses conducted by several researchers [43,44,45] confirmed the presence of different groups of pharmacologically active chemical compounds in *M. oleifera*. These chemical compounds included: alkaloids, glycosides, flavonoids, phenols, phytosterols, saponins, tannins, coumarins and terpenoids. In addition, functional compounds of nutritional value such as carbohydrates, proteins, fats, and vitamins have also been discovered [46]. The contents of proteins (27.2%) and fats (17.1%) in *M. oleifera* leaves was lower than that observed in other plant parts such as seeds [43]. The mean dried leaves crude protein content was 28.7–30.3% and consisted of 19 amino acids [8]. A protein contents (on dry basis) of 25.1–29.0% was also reported in dried leaves [44]. Whereas *M. oleifera* seeds proteins and fats contents were reported to be 34% and 33.23%, respectively [45]. The high protein content of *M. oleifera* leaves makes it a good potential supplementary protein source in animal feed. Other studies had recorded various protein contents that ranged between 16% and 40% [47,48,49,50,51,52].

The oil content of *M. oleifera* seeds ranges from 25–42% [40] to 75% [53]. Seventeen fatty acids were observed, with α-Linolenic acid (44.57%) having the highest value followed by eicosanoid (14.41%), g-linolenic (0.20%), palmitic (0.17%) and capric acid (0.07%) [8]. Vitamin E had the highest concentration of 77 mg/100 g, while beta-carotene concentration was 18.5 mg/100 g in the dried leaves [8]. The oil is bright yellow in color and has good oxidative stability that makes it edible [53]. The oil is used as a lubricant for precision machinery because it does not tend to deteriorate or become rancid and viscous [54]. It is also useful as a vegetable oil for cooking. The oil is characterized by its ability to absorb and retain volatile substances, so it is valuable in the perfume industry to stabilize scents. The content of the free fatty acids ranges between 0.5–3%. *M. oleifera* leaf extract using 80% ethanol contains growth stimulants (such as cytokins). This extract can be used as a foliar spray to promote the growth of young seedlings and using a growth hormone spray will make the plants more durable, resistant to pests and diseases and will produce more and larger fruits at the harvest stage [55].

The contents of crude fiber (19.4%) and ash (11.1%) in *M. oleifera* dried leaves were higher [43] compared to those of Anwar and Mohammed [45], who recorded values for crude fiber (7.5%) and ash (7%) in *M. oleifera* seeds. The dried *M. oleifera* leaves had mineral contents: Ca (3.65%), P (0.3%), Mg (0.5%), K (1.5%), Na (0.164%), S (0.63%), Zn (31.03 mg/kg), Cu (8.25%), Mn (86.8 μg/kg), Fe (490 μg/kg) and Se (363 μg/kg) [8]. The fiber content of dried *M. oleifera* leaves consisted of 11.4% NDF (Neutral detergent fiber), 8.49% ADF (Acid detergent fiber), 1.8% ADL (Acid detergent lignin), 4.01% ADC (Acid detergent cellulose) and 3.2% tannins, while the total polyphenols constituted 2.02% [8]. The values of amino acids, fatty acids, minerals and vitamin profiles reflect a desirable nutritional balance. On the other hand, *M. oleifera* contains some antinutrient substances such as hydrogen cyanide, oxalate and phytate which could limit its uses as feed [46,56].

## 3. Overview of the Use of *Moringa oleifera* in Feeding of Livestock Animals

Economic promotion and human health in developing countries can be enhanced by research on animal production, particularly by research on nutritionally valued and relatively cheap nonconventional feedstuffs. The proximate analysis of *M. oleifera* leaves conducted in our laboratory compared to that of alfalfa hay is shown in Table 1.

### 3.1. Milk Production

The use of *M. oleifera* leaves or whole plants as feed components to enhance milk production is attracting researchers’ attention due to the high nutritional value of *M. oleifera* leaves [57], which is crucial for the improvement of milk yield and quality in ruminants [58]. *M. oleifera* leaves are also known for their high protein contents and can therefore boost microbial protein synthesis in the forestomaches of ruminants [59].

Many researchers dealt with the partial or complete replacements of feedstuffs with *M. oleifera* to improve milk production in goats, sheep and cows [52,58,60,61,62,63]. Our previous studies conducted on goats and ewes by partial replacement (62.5%) of alfalfa hay with *M. oleifera* leaves revealed higher milk yield and milk fat, lactose, solid-non-fat and energy contents in both goats and ewes [58]. The milk of goats and ewes fed on rations in which alfalfa hay was partially replaced by *M. oleifera* leaves also showed higher catalase and vitamin C levels and reflected higher milk oxidative stability compared to those fed on alfalfa hay-based ration [61]. On the other hand, partial replacement (50%) of concentrate mixture with *M. oleifera* leaves resulted in higher milk yield and milk fat, protein, lactose and solid-non-fat contents in lactating Bengal goats [60]. The improvement of milk quality and yield due to inclusion of *M. oleifera* leaves in rations could be attributed the rich micronutrients contents of *M. oleifera* leaves compared to alfalfa (Table 2).

Replacement of *Trifolium alexandrinum* hay with *M. oleifera* leaves in the ration of creole dairy cows resulted in higher dry matter intake and milk yield [52]. In another study, alfalfa was partially (50%) or completely (100%) replaced with *M. oleifera*, where cows fed on an *M. oleifera* based diet showed higher milk yield compared to those fed on an alfalfa-based diet [61]. Further *M. oleifera* based rations enriched solid and solid-non-fat, protein, and ash contents of cow’s milk compared to alfalfa-based rations [61]. On the other hand, replacement of cotton seed cake with *M. oleifera* leaves meal at 10, 20 or 30% dry matter content in dairy cows’ ration improved milk yield without significant effects on milk composition [52]. Furthermore, mixing *M. oleifera* leaves with chopped wheat hay and sugar cane molasses at a ratio of 370:540:90 on dry matter basis, and the addition of this mixture at a rate of 180 g/kg dry matter as a replacement for wheat silage and hay to the total mixed ration (TMR) of lactating cows, resulted in higher milk yield and milk fat content despite the reduction of digestible dry matter intake compared to the control group [62]. However, the limited replacement (≤50%) of alfalfa hay and maize silage with *M. oleifera* silage did not influence the milk yield or serum biochemical profile of lactating Holstein cows [63]. The reported improvement of milk quality and yield due to inclusion of *M. oleifera* leaves in ruminants’ rations could be attributed to its promoting effects on rumen microbes [52] as well as its good rumen bypass attributes, which is crucial for milk production [50].

### 3.2. Meat Quality and Production

*M. oleifera* has been recommended as a supplementary feed for dairy cows, goats and fish since it contains high levels of crude protein in the leaves [52,64]. Moreover, it has high levels of antioxidant compounds [65], which could modulate meat quality [66].

In a study by Moyo et al. [67], crossbred Xhosa lop-eared goats given *Moringa oleifera* leaf meal showed chevon with the highest physiochemical traits and consumer sensory effects when compared to grass hay and sunflower seed cake. The meat of Moringa-fed goats also showed the lowest shearing and cooking loss values than the other two treatments. The improved tenderness in the Moringa-fed group was ascribed to the increased levels of intramuscular fat in the animals. Another study by Sultana et al. [68] showed that adding *M. oleifera* to goats’ diets had resulted in meat with higher ratios of polyunsaturated fatty acids, decreased ratio of n-6/n-3 and reduced lipid oxidation. The authors also cited the active components of Moringa leaves as phenolic, flavonoids and carotenoids which have been proven to contain significant antioxidant potential, where polyphenols can protect meat from lipid oxidation by acting as a chain-breaking proxy-radical scavenger. Melesse et al. [69] studied the effect of air-dried *M. stenopetala* leaves supplementation on the carcass and meat quality characteristics of goats. Their results revealed that animals in supplemented groups performed better than unsupplemented ones with statistically enhanced slaughter weight, daily weigh gain, rib-eye area and dressing percentage. Although meat cooking loss was not significantly different between the treatment groups, the supplemented groups showed improved cooking loss compared to the control. Adegun et al. [70] reported that *M. oleifera* can enhance the performance of sheep as protein supplements. The same authors also cited that supplementation of Moringa-based multi-nutrient blocks in sheep’s diet enhanced performance and pose no health challenges to the animals. Moreover, animals in the Moringa group attained the highest weight gain compared to the other treatment groups. Adding *Moringa oleifera* silage to Assaf lambs’ diet is also proven to produce a tender and lean lamb meat [71]. In this study, the meat of Moringa-fed lambs was more tender compared to the control and characterized by longer sarcomere and lower intra-muscular fat content.

Nkukwana [72] studied the effects of dietary supplementation with vitamin E and *M. oleifera* ground leaves on broiler chickens’ meat quality. He concluded that the dietary supplementation significantly improved the broiler meat quality. The body weight gain and carcass yield were improved with increased inclusion of *M. oleifera* ground leaves. In addition, Nduku et al. [73] reported that the effects of *M. oleifera* leaf meal were comparable with probiotic and organic acid in improving growth performance, digestive organ size and meat quality characteristics when included in broiler diets as an alternative to growth promoters. The birds fed on *M. oleifera* leaf meal showed significant increase in meat redness. In a study that tested feeding *M. oleifera* leaf meal to chicken, carcass characteristics were significantly affected in diets containing *M. oleifera* leaf meal, as well as sensory evaluation parameters such as appearance, flavor, tenderness, juiciness and palatability, which were highly correlated with the increasing level of *M. oleifera* leaf meal [74]. Mardewi et al. [75] cited that supplementation of *M. oleifera* powder in chicken rations increased the quality of meat without decreasing the weight percentage of the carcass. Moreover, the subcutaneous fat and cholesterol contents decreased in the supplemented groups. Interestingly, El Tazi [76] mentioned that hot and cold carcass weight, dressing percentage, breast and drumstick percentages, as well as the tenderness and juiciness scores of both breast and thigh meat, were statistically improved due to *M. oleifera* leaf meal inclusion. On the other hand, Sebola et al. [77] fed *M. oleifera* leaf meal to different chicken strains and evaluated the quality and fatty acid profile of the meat. They concluded that using *M. oleifera* leaf as a feed supplement for chicken resulted in improved meat tenderness but did not alter the fatty acid profile.

### 3.3. Poultry Production

Poultry production in many countries is facing a lack of high-quality feed with the rising cost of poultry feed due to the lack of expensive raw ingredients, especially proteins [78]. The competition between humans and livestock for the same products available for feed is exacerbating the situation, thus there is a need to obtain other sources of available low-cost feed ingredients that would replace the existing raw ingredients, especially soybeans and fishmeal.

Feed is an integral part of poultry production which accounts for about 70–80% of the production cost and at the same time affects the level and quality of production depending on the feeding regime and the quality of the feed [79]. In most developing countries, the primary protein sources in commercial poultry feeds are fishmeal and soybeans. However, these ingredients are production constraints in the poultry industry; they are usually scarce and expensive and are widely used by livestock breeders and in human nutrition. Thus, there is a need to search for unconventional, cheap, locally available and less competitive plant protein sources as alternative protein sources in poultry diets. Legume crops have been suggested as alternative proteins, vitamins and minerals sources in poultry diets [80].

*M. oleifera* can replace traditional feed because of its beneficial properties [47]. There is an increasing popularity of using *M. oleifera* as a feed additive in poultry feed; however, further research should be conducted in order to fully understand its nutritional value and its effect on blood immunological parameters as a measure of both the nutritional and medicinal benefits of the leaves on broiler chicks [81].

The use of *M. oleifera* in poultry rations has an important physiological function due to the antioxidant effect of some of its compounds [82], which protects poultry from the harmful effects of oxidation [83]. There are also many different ideas about both the proportions and the part of the plant used, i.e., whether leaves or seeds. Many previous studies reported that *M. oleifera* might have a positive role in improving the productive performance and health status of poultry [84]. However, more studies are still needed to discover the actual dietary inclusion levels for the optimum performance in poultry.

Special care must be taken to avoid excessive protein consumption when utilizing fresh Moringa plant parts [85]. The plant parts of Moringa, in their raw form, appear to reduce the activity of pathogenic bacteria and molds and improve the digestion of other feed components, thus helping poultry to express their genetic potential [86]. It has been reported that the inclusion *M. oleifera* leaf extract in feed at a concentration of 24% had no adverse effect on live body weight, mean daily weight gain, feed conversion ratio (FCR), mortality, carcass and organ characteristics in poultry as compared to the control meal [87].

*M. oleifera* leaves contain essential amino acids, especially sulfur amino acids, whose concentrations are higher than those recommended by the Food and Agriculture Organization (FAO) and similar to those of soybean seeds [83]. *M. oleifera* leaves are suitable feed ingredients for poultry, since they have low trypsin inhibitors and tannins content that may affect the normal digestion and metabolism of nutrients in poultry [88]. Moreover, they contain trace levels of acidic proteins with hemagglutinating activity (65.5 μmol/g) and phytates (41 g/kg). Phytates (1–6%) reduce the bioavailability of minerals in poultry, especially Zn^+2^ and Ca^+2^. These excellent nutritional properties make it suitable as animal feed [86]. Therefore, *M. oleifera* leaves are a promising protein source used in poultry diets at low levels [89].

Many studies have shown that the nutritional content of *M. oleifera* leaves on a dry matter (DM) basis is 17.01–29.7% crude protein, 63.11–69.40% carbohydrates, 4.38–21.09% crude fiber, 2.11–6.41% raw fat, 7.96–8.40% Ash, 14.790 MJ/kg gross energy and 1.69–29.9% ether extract [48,86,90]. Briones et al. [91] reported that *M. oleifera* leaves can be used as a nutritional supplement in laying hens and broiler chicks’ feed due to its positive effects on performance production and egg quality. However, there are still debates on the inclusion levels of *M. oleifera* necessary to have a positive effect on chickens’ performance. *M. oleifera* also contains vitamins such as beta-carotene, folic acid, pyridoxine, nicotinic acid and vitamins C, D and E [5]. The nutritional value of *M. oleifera* leaves for poultry can be increased by a phytase enzyme which breaks down phytates and leads to an increase in phosphorous absorption. In such cases, phytase should be mixed with the leaves without heating [92].

The use of fresh *M. oleifera* leaves in feeding broiler chickens (ad libitum) did not affect body weight gain; however, the net income per bird increased by $0.161. Accordingly, *M. oleifera* leaves and seeds can be used efficiently as a feed supplement in laying hens and broiler chicks’ diets to improve feed utilization efficiency [91]. In addition, the use of *M. oleifera* leaves in poultry feed improved the growth performance of chicks compared to maize alone [93].

The use of *M. oleifera* leaves at a ratio of 15 and 20% in poultry feed increased chicks’ body weight. However, supplementation of *M. oleifera* leaves at 5 and 10% in broiler chicks did not impact the body weight of broiler chicks [94]. The effect of including *M. oleifera* leaves in a cassava flake-based diet of laying hens was evaluated by Olugbemi et al. [95]. They found that the inclusion of *M. oleifera* leaves did not affect egg production performance. However, the cost of feed per kilogram of eggs produced has been reduced due to dietary inclusion of *M. oleifera* leaves. Moreover, the acceptance of cooked eggs was higher in the group which received 10% *M. oleifera* leaves in their diet. Furthermore, Ashong and Brown [90] reported no signs of abnormal behavior, toxicity and mortality among White Leghorn chicks fed diets supplemented with *M. oleifera* leaves. They also demonstrated the feasibility of integrating *M. oleifera* leaves in growing poultry diets.

The supplementation of Commercial broilers’ diets with *M. oleifera* leaves extract increased broilers body weight, reduced feed intake and consequently improved feed utilization efficiency compared to unsupplemented diets [96]. However, in another study, Gadzirayi [97] did not report any variation in feed intake between poultry fed *M. oleifera* leaves extract-supplemented and unsupplemented diets, whereas Portugaliza and Fernandez [98] added different percentages of *M. oleifera* leaves extract to broiler’s drinking water and claimed that addition of 30, 60 or 90 mL of the extract per liter of drinking water reduced feed consumption and increased live body weight and the efficiency of converting feed to meat. Similarly, dietary inclusion of *M. oleifera* leaves extract at 0.5% improved broilers growth performance, carcass yield and economic returns [99]. The same author also claimed that *M. oleifera* leaves extract can adequately replace expensive protein sources in broiler diets, reducing production costs without compromising performance.

The possibility of using *M. oleifera* leaves extract as a source of protein at a rate of 10% in laying hens’ diets has been reported [100]. The same authors further claimed that, at higher prices of protein sources, *M. oleifera* leaves extract can comprise up to 20% of poultry feed. In contrast, the *M. oleifera* leaves meal reduced egg yield, egg mass and feed intake when supplemented to the diet at the rate of 15% [101]. Moreover, it has been indicated that average live body weight and average daily weight gain were significantly decreased, while the feed conversion ratio was significantly improved in broiler chickens supplemented with *M. oleifera* leaves extract at 90–120 mL/L drinking water [102]. Recently, Abubakar et al. [103] showed that diet supplementation with *M. oleifera* leaves extract resulted in the improvement of egg-laying performance as well as of the internal and external egg quality characteristics of laying hens. Meanwhile, no adverse effects were observed concerning shell weight, shell thickness and egg shape index, which are important characteristics for egg transportation and handling [100]. The inclusion of *M. oleifera* leaves extract up to 10% has been accepted in feed for Rhode Island chickens, as higher levels reduced the rate of egg production [104].

## 4. Conclusions and Future Prospects

Moringa oleifera is widely known as a source of food, fodder, medicine, plant growth stimulants, cosmetics, water purification and biofuel production. The reports in the reviewed literature particularly encourage the use of *M. oleifera* whole plants as nonconventional forage in ruminants’ nutrition, as well as the use of *M. oleifera* leaves or leaves extract as a protein source for broilers and laying hens. However, further research is needed to determine the appropriate supplementation level of different plant parts or their extracts, as well as the appropriate processing methods or treatments of *M. oleifera*, in order to improve its palatability and consequently enhance the production performance of livestock without compromising animal health.

The inclusion of *M. oleifera* in the feed of livestock animals with the ultimate goal of producing functional food (meat, eggs and milk) with appropriate contents of human health-promoting substances such as omega 3 and organic selenium remains to be elucidated. Furthermore, *M. oleifera* inclusion in livestock diet has the potential to increase the shelf life of animal products during storage and processing. Detailed investigation of these criteria in the prospective research would satisfy producer and consumer demand in this niche market. Finally, it is crucial to conduct cost–benefit studies on the use of *M. oleifera* as feed stuff for livestock in future research.

## Figures and Tables

**Table 1:** The proximate analysis of *M. oleifera* leaves and alfalfa hay (% DM basis) ^1^.

Nutrient	*M. oleifera* Leaves	Alfalfa Hay
Crude protein	28.97	17.60
Crude fat	7.97	01.60
Crude fiber	7.57	29.40
Ash	10.87	09.63

^1^ Modified from Babiker et al. [15] and Mohamed-Ali et al. [16].

**Table 2:** Some macro- and micronutrients contents in *M. oleifera* leaves compared to alfalfa hay ^1^.

Nutrient	*M. oleifera* Leaves	Alfalfa Hay
P (%)	0.33	0.27
Ca (%)	2.47	1.53
Mg (%)	0.41	0.45
K (%)	1.89	1.49
Fe (ppm)	546.90	357.00
Cu (ppm)	5.00	12.00
Zn (ppm)	34.30	22.00
Mn (ppm)	51.90	43.00
Se (ppm)	0.60	0.07

^1^ Modified from Hall et al. [14] and Babiker et al. [15].

## Data Availability

Not applicable.
